# Mid-Term Feasibility of Percutaneous Left Atrial Appendage Occlusion in Elderly Patients with Non-Valvular Atrial Fibrillation

**DOI:** 10.3390/jcm12186024

**Published:** 2023-09-18

**Authors:** Nobuyuki Fukuda, Teruhiko Imamura, Shuhei Tanaka, Naoya Kataoka, Ryuichi Ushijima, Hiroshi Ueno, Koichiro Kinugawa

**Affiliations:** The Second Department of Internal Medicine, University of Toyama, Toyama 930-8555, Japan; nfukuda@med.u-toyama.ac.jp (N.F.); nkataoka@med.u-toyama.ac.jp (N.K.); kinugawa-tky@umin.ac.jp (K.K.)

**Keywords:** atrial fibrillation, anticoagulation, thrombosis

## Abstract

Background: Percutaneous left atrial appendage occlusion (LAAO) therapy using the WATCHMAN system has been introduced to prevent thrombosis and minimize the use of anticoagulants in patients with non-valvular atrial fibrillation. Given the high risk of bleeding and stroke in elderly patients, these patients would be good candidates for this therapy. However, the efficacy and feasibility of LAAO therapy in elderly patients remains uncertain. Methods: Consecutive patients who underwent LAAO therapy using the WATCHMAN system in a large academic center between June 2020 and March 2023 were included. The safety and efficacy of LAAO therapy during the 1-year observation period in patients aged ≥85 years old were compared with those in the younger cohort. Results: A total of 188 patients (78.4 ± 6.9 years old, 133 male patients) were included. 34 patients were ≥85 years old, 96 were between 75 and 84 years old, and 58 were <75 years old. The elderly group had a higher CHA_2_DS_2_-VASc score and were at greater risk of falling-related bleeding compared with the younger cohort. The device implantations were successful in all patients except for one. During the 1-year observation period, one patient had a peri-device leak >5 mm and there were 6 device-related cases of thrombosis, whose incidence was not significantly different between the groups (*p* = 0.98). The cumulative incidences of bleeding and thrombotic events in the elderly group were as low as in the younger cohort (*p* > 0.05 for both). Most anticoagulants were terminated regardless of age. Conclusion: The mid-term feasibility and efficacy of percutaneous LAAO therapy using the WATCHMAN system in elderly patients aged ≥85 years were as acceptable as in the younger cohort.

## 1. Background

The number of patients with non-valvular atrial fibrillation (NVAF) has been increasing as the population ages [1]. Elderly patients with NVAF have an incremental risk of stroke, while they also have an incremental risk of falling-related major bleeding [2,3]. Thus, anticoagulation therapy in elderly patients with NVAF poses a therapeutic dilemma [4,5,6,7,8]. The recently introduced direct oral anticoagulant (DOAC) is superior to conventional warfarin but has not yet completely resolved the therapeutic dilemma. In addition, the addition of a DOAC in elderly patients presents several problems related to polypharmacy and adherence [9].

Most of the cases of intra-cardiac thrombus originate from the left atrial appendage (LAA) [10]. Several percutaneous LAA occlusion (LAAO) devices have been innovated to suppress the formation of thrombus in LAA [11,12,13]. In the PROTECT AF and PREVAIL trials, the WATCHMAN system was non-inferior to warfarin in preventing stroke in patients with NVAF and a high-risk of bleeding [11,12]. The device was non-inferior to the DOAC in the PRAGUE-17 trial [13].

The rates of procedural success and procedural complication improved in the EVOLUTION registry [14]. The feasibility of the WATCHMAN system was further enhanced by the system innovation from WATCHMAN 2.5 to WATCHMAN FLX [15,16]. In light of the SALUTE trial, which demonstrated the feasibility of the WATCHMAN system in 42 Japanese patients, the device was approved by the Japanese government in 2019 [17].

Clinical outcomes of LAAO therapy in real-world clinical practice have been reported, while those of the elderly cohort at high risk of stroke/bleeding remain uncertain. In this retrospective study, we evaluated the feasibility of LAAO therapy in the real-world elderly cohort.

## 2. Methods

### 2.1. Patient Selection

We prospectively included consecutive patients with NVAF who underwent percutaneous LAAO therapy using the WATCHMAN system from June 2020 in a large academic center in our registry database. We conducted this study retrospectively using this dataset. We used the WATCHMAN 2.5 until June 2021 and converted the system to WATCHMAN FLX in June 2021. We included patients who underwent LAAO therapy until March 2023. All patients gave statements of informed consent to be included in our registry. The ethics committee of our center approved the study protocol (R2020077).

### 2.2. Indication of LAAO

The indication of LAAO was determined according to the guidelines of The Japanese Society of Cardiology [18]. Patients with NVAF who were at high risk of stroke according to CHADS_2_ scores and CHA_2_DS_2_-VASc scores were eligible for LAAO therapy [19]. Patients who had a high-risk of bleeding with a HAS-BLED score of ≥3 points, those who had a history of repeated falling, those with diffuse cerebral amyloid angiopathy, those who required multiple antiplatelets for over 1 year, and those with a history of major bleeding assigned to BARC type 3, were eligible. Patients who were contraindicated to anticoagulation were also considered to be eligible. The final indication was determined by the multidisciplinary heart and valve team.

### 2.3. Procedure

Percutaneous LAAO therapy was performed under general anesthesia using angiography and transesophageal echocardiography supports, according to the standard procedure. All procedures were performed by the two proctor-certified operators. Intra-procedure echocardiography was performed by the board-certified echocardiologist who had received a specific training program for the procedure.

### 2.4. Post-Procedure Management

Post-procedural antithrombotic therapy was administered according to the recommended regimen. Following the procedure, anticoagulation therapy, using warfarin or a DOAC, and antiplatelet therapy, using one of three agents (aspirin, clopidogrel, and prasugrel), were performed for 45 days, after which transesophageal echocardiography was performed. Then, anticoagulation therapy was terminated, and dual antiplatelet therapy was initiated, unless a major para-device leak or device-related thrombus was observed. Dual antiplatelet therapy was downgraded to single antiplatelet therapy six months later. The detailed medical therapy regimen was adjusted at the discretion of the attending physicians, considering the risks of bleeding and thrombosis.

### 2.5. Study Outcome

All patients were stratified into three groups according to their age: <75 years, 75–84 years, and ≥85 years. Clinical outcomes as detailed below were compared between the three groups.

Procedure-related events were counted during the procedure and for 1-week post-procedure, or until the index discharge. Procedure-related events were defined as death, cerebrovascular events, systemic embolism, air embolism, bleeding, pericardial effusion, device embolization, and acute kidney injury. Of these, major procedure-related events were death, cerebrovascular events, systemic embolism, bleeding assigned to BARC 3–5, relevant pericardial effusion, device embolization, and acute kidney injury.

Mid-term clinical events were counted at 45 days, 1 year, and over 1 year, including death, cardiovascular death, cardiovascular event, systemic embolism, bleeding, myocardial infarction, ischemic stroke, hemorrhagic stroke, transient ischemic attack, and bleeding.

A performance target of acute event rates was defined as <10% according to the SALUT trial. Another performance target of LAA closure with a peri-device leak < 5 mm was defined as >94%, also according to the SALUTE trial [17].

### 2.6. Statistical Analysis

Continuous variables were expressed as a mean and standard deviation and compared between the three groups using an analysis of variance. Categorical variables were expressed as numbers and percentages and compared between the three groups using Fisher’s exact test. Cumulative incidences of the clinical events were compared between the three groups using log-rank tests. A value of *p* < 0.05 was considered statistically significant. Statistical analyses were performed using SPSS Statistics 24 (SPSS Inc., Armonk, NY, USA).

## 3. Results

### 3.1. Baseline Characteristics

A total of 188 patients were included (Table 1). Age was 78.5 ± 6.8 years old and 54 (29%) were female patients. All patients had NVAF. The CHADS_2_ score was 3.5 ± 1.3 and HAS-BLED score was 2.9 ± 1.0. 34 (18%) patients had a high risk of bleeding due to falling and 96 (51%) had a history of relevant bleeding. Most of the patients had received single anticoagulation therapy or single anticoagulation and single antiplatelet therapy.

58 patients were <75 years old, 96 patients were between 75 and 84 years old, and 34 patients were ≥85 years old. A higher age was associated with an incremental CHADS_2_ score (*p* < 0.01). The HAS-BLED scores were lower in patients with older age, whereas the prevalence of high risk for falling-related bleeding was higher in the elderly patients (*p* < 0.01 for both).

### 3.2. Procedure Data

**Figure 1:**
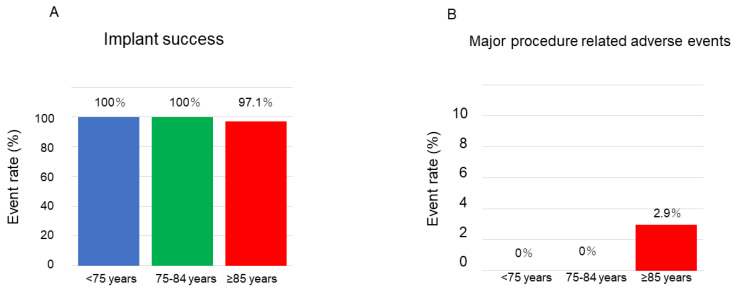
Procedure success rate and major adverse event rate. (**A**) WATCHMAN implant success rates stratified by age. (**B**) Major adverse events within 7 days of procedure stratified by age. Event rates were compared between the groups using Fisher’s exact test.

All procedures were successful under general anesthesia with a transesophageal echocardiography guide, except for one patient, in whom the LAA size was too large to place the device (Figure 1A). The WATCHMAN 2.5 was implanted in 48 patients and the WATCHMAN FLX was implanted in 139 patients (Table 2). The most prevalent size of the WATCHMAN 2.5 was 33 mm and that of the WATCHMAN FLX was 31 mm. No patients had a peri-device leak >5 mm. Most patients (72%) received an anticoagulant and single antiplatelet at index discharge.

Procedure-related parameters were not significantly different between the groups, including the procedure time and contrast amount (*p* > 0.05). The prevalence of medication type was not significantly different between the groups (*p* > 0.05 for all).

### 3.3. Mid-Term Echocardiographic and Medication Follow-Up

Transesophageal echocardiography was performed in most patients at the 45-day follow-up (Table 3). A peri-device leak and device-related thrombosis were observed in one patient with the WATCHMAN 2.5. Anticoagulant therapy was terminated in >80% of patients regardless of age group (*p* > 0.05).

**Figure 2:**
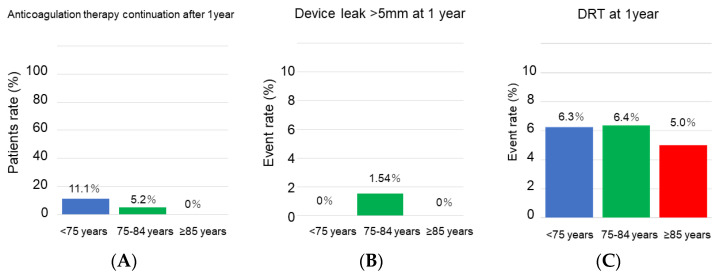
Medication and complication at 1-year follow-up. (**A**) The rate of anticoagulation therapy. (**B**) The rate of device leak > 5 mm. (**C**) The rate of DRT, DRT, device related thrombus. Event rates were compared between the groups using Fisher’s exact test.

**Figure 3:**
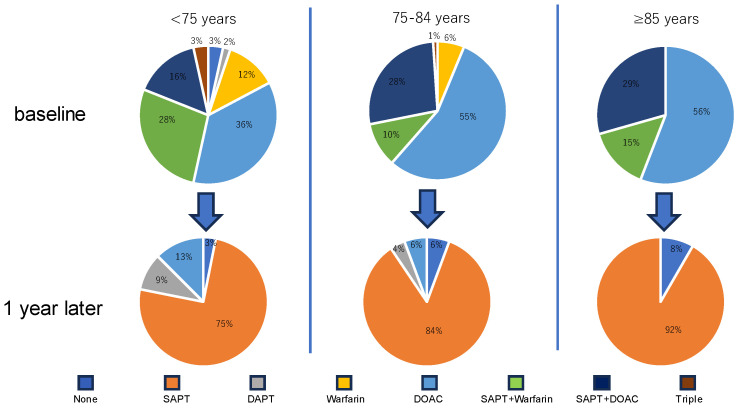
Trajectory of medication between baseline and 1-year follow-up. SAPT, single antiplatelet therapy; DAPT, dual antiplatelet therapy; DOAC, direct oral anticoagulant, Triple, DAPT and anticoagulant therapy.

Transesophageal echocardiographic and medication data at the 1-year follow-up are displayed in Table 4 and Figure 2. Only one patient had a peri-device leak > 5 mm. Six patients had device-related thrombosis, and its incidence was not significantly different between the groups (*p* = 0.98). Only seven patients (6%) continued anticoagulation therapy, and its prevalence was not significantly different between the groups (*p* = 0.18). Detailed trajectory of medication is displayed in Figure 3. All patients who were ≥85 years old terminated anticoagulation therapy.

### 3.4. Clinical Events

Table 5 summarizes the clinical outcomes after the procedure. There were two procedure-related events: progression of anemia that required trans-infusion and minor pericardial effusion. In the patient who required blood transfusion, there was no intra-procedural pericardial effusion, no bleeding at the puncture site, and no progression of anemia. The patient had heart failure, and 4 days after the procedure, a blood transfusion was performed to further improve anemia. No patients had device migration or device embolization. There were two procedure-unrelated events: sudden death due to myocardial infarction and gastrointestinal bleeding that required trans-infusion. A patient died suddenly at home 5 days after the procedure. The autopsy demonstrated that he died due to atherosclerosis-related acute myocardial infarction. The event rates were not significantly different between the groups (*p* = 0.10; Figure 1B).

**Figure 4:**
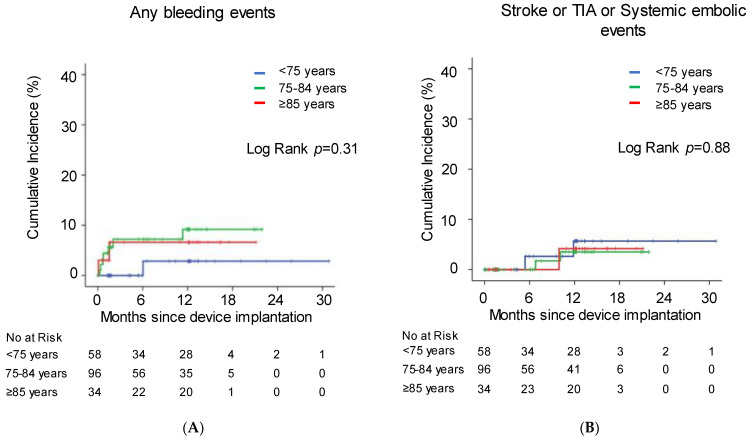
Cumulative incidence of any bleeding events (**A**) and thrombosis-related events (**B**) stratified by age. Kaplan–Meier curves were compared between the groups using log-rank test.

Patients were followed for 280 ± 181 days after index discharge. There were thirteen deaths, consisting of three malignancies, two cerebrovascular diseases, two cases of pneumonia, two cases of renal failure, one case of heart failure, one myocardial infarction, and two of unknown origin. There were five embolism-related events and ten cases of bleeding. There were no significant differences in the incidence of these events between the groups (*p* > 0.05 for all). Cumulative incidence of bleeding and embolism-related events were not significantly stratified by age (*p* > 0.05 for both; Figure 4A,B).

**Figure 5:**
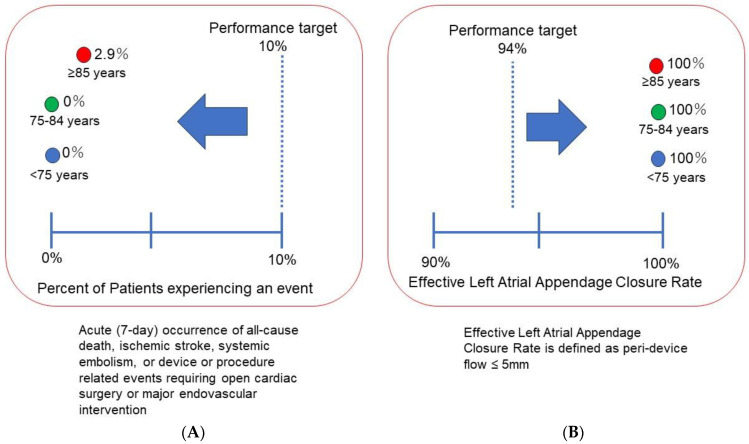
The rate of acute events (**A**) and effective LAAO defined as peri-device flow ≤ 5 mm (**B**) in each age-stratified group. Performance target was recommended according to the findings of SALUTE trial.

Acute event rates were within 10%, which was a performance target defined according to the SALUT trial, regardless of age (Figure 5A). The rates of effective LAA closure with a peri-device leak < 5 mm, were over 94%, which was also a performance target defined according to the SALUT trial, regardless of age (Figure 5B).

## 4. Discussion

In this retrospective study conducted in a single academic center using prospectively collected registry data, we compared clinical outcomes between elderly patients aged ≥85 years and others. Percutaneous LAAO therapy using the WATCHMAN system was effective and feasible even in the elderly patients who had high a CHADS-VASc score and HAS-BLED score. Anticoagulation therapy could also be terminated in this elderly cohort without increasing thromboembolic events.

### 4.1. Clinical Implication of LAAO in the Elderly Patients with NVAF

Anticoagulation therapy is a gold standard therapy for patients with NVAF to prevent stroke [19]. Elderly patients have an incremental incidence of NVAF and greater risk of stroke due to multiple risk factors [20]. Nevertheless, they have a higher risk of falling due to increasing frailty [21]. The issue of polypharmacy is particularly evident in this cohort [22]. Furthermore, they have a higher risk of bleeding [3]. Although the DOAC has been introduced as a superior alternative to conventional warfarin, the DOAC cannot completely overcome the above dilemma in the elderly patients with NVAF, who have higher risk of both bleeding and stroke [23].

Thus, the less invasive and effective LAAO therapy has been receiving great attention as a good alternative to the DOAC in elderly patients with NVAF.

### 4.2. Device Innovation

The 1-year clinical efficacy of the WATCHMAN 2.5 was demonstrated by the EVOLUTION trial and NCDR registry [24,25]. The mid-term clinical outcome of the next generation WATCHMAN FLX was shown in the PINACLE FLX trial and FLXibility trial. Our team also demonstrated the comparable feasibility of the WATCHMAN FLX to the WATCHMAN 2.5 [26]. Thus, we combined clinical data of both devices in this study. In this study, the procedure success rate was 100% in patients receiving the WATCHMAN FLX, and procedure-related complications in the WATCHMAN FLX therapy was 0%. WATCHMAN FLX would be a more suitable device for elderly patients.

### 4.3. Safety of LAAO in the Elderly Patients

Percutaneous interventions are generally less invasive than the surgical approach and would be suitable for elderly patients, since such patients require particularly safe approaches in comparison to younger patients. Several procedure-related complications have been reported, including thromboembolism, stroke, cardiac tamponade, and vascular injury. The incidence of these complications has decreased considerably due to procedural improvement and device innovation [15,16,24,25].

In this study, the feasibility of LAAO therapy was shown to be comparable to that of recent large-scale studies [15,16]. It is noteworthy that the procedure was feasible regardless of high age. High age may not be a contraindication of LAAO therapy, although further studies are warranted for optimal patient selection.

### 4.4. Efficacy of LAAO in the Elderly Patients

Elderly patients have higher risks of both bleeding and stroke [9,23]. Theoretically, they are rather good candidates for LAAO therapy, since this therapy decreases the risk of bleeding and stroke by terminating the use of anticoagulants. In our study, the stroke rate in the elderly patients was as low as that of the younger cohort, whereas the use of anticoagulants could be terminated in most elderly patients safely at a comparable level to the younger cohort.

### 4.5. Medication after LAAO

Anticoagulation and antiplatelet therapy were performed according to the guidelines, in principle, whereas a few studies reported the feasibility of a non-anticoagulation regimen [27]. Several risk factors of device-related thrombosis, which is one of the critical complications, have been reported, including spontaneous echo contrast in the left atrium, and peri-device leak [28]. However, the detailed mechanism of device-related thrombosis remains uncertain, and repeated follow-ups using echocardiography and/or computed tomography would be essential to adjust medication. In this study, anticoagulation, which is associated with the risk of bleeding, could be terminated in elderly patients.

### 4.6. Limitations

The patients’ number was moderate. It is noteworthy that the sample size of elderly patients aged ≥85 years old was small. We demonstrated non-inferiority in the clinical outcomes of the elderly patients compared to the younger patients, but statistical non-significance does not necessarily indicate similarity between the groups, particularly in a small sample sized study with a low event rate. The observation period was mid-term, given the recent availability of LAAO therapy. Further long-term multi-center studies are warranted to validate the efficacy and feasibility of LAAO therapy in elderly patients. We believe that the present study could be a proof-of-concept for larger studies including the target population from a variety of geographic areas.

## 5. Conclusions

The feasibility and efficacy of percutaneous LAAO therapy using the WATCHMAN system in elderly patients with NVAF were as high as those of the younger cohort. Long-term multi-center studies are warranted to validate our findings.

## Figures and Tables

**Table 1:** Baseline characteristics.

	Total (N = 188)	<75 Years (N = 58)	75–84 Years (N = 96)	≥85 Years (N = 34)	*p* Value
Demographics					
Sex, female	54 (29)	11 (19)	24 (25)	19 (56)	<0.01 *
Age (years)	78.5 ± 6.8	70.8 ± 4.0	79.7 ± 2.9	88.1 ± 2.6	<0.01 *
Body mass index (kg/m^2^)	23.2 ± 3.8	24.0 ± 3.9	22.9 ± 3.7	22.6 ± 3.8	0.13
Body surface area (m^2^)	1.59 ± 0.17	1.67 ± 0.16	1.59 ± 1.57	1.45 ± 0.15	<0.01 *
Comorbidity					
Heart failure	124 (66)	35 (60)	61 (64)	28 (82)	0.08
New York Heart Association class II–IV	114 (61)	30 (52)	58 (60)	26 (76)	0.06
Hypertension	146 (78)	46 (79)	74 (77)	26 (76)	0.93
Diabetes mellitus	59 (31)	21 (36)	30 (31)	8 (24)	0.45
Prior stroke or transient ischemia attach	94 (50)	32 (55)	47 (49)	15 (44)	0.57
Prior ischemic stroke	70 (37)	22 (38)	35 (36)	13 (38)	0.98
Prior hemorrhagic stroke	31 (16)	11 (19)	13 (14)	7 (21)	0.77
Prior transient ischemic attack	13 (7)	3 (5)	9 (9)	1 (3)	0.37
Prior thromboembolic events	21 (11)	12 (21)	7 (7)	2 (6)	0.021 *
Hyperlipidemia	110 (59)	33 (57)	58 (60)	19 (56)	0.86
Coronary artery disease	91 (48)	29 (50)	46 (48)	16 (47)	0.96
Chronic obstructive pulmonary disease	7 (4)	1 (2)	6 (6)	0 (0)	0.16
Peripheral arterial disease	14 (7)	8 (14)	5 (5)	1 (3)	0.08
Chronic dialysis	35 (19)	21 (36)	12 (13)	2 (6)	<0.01 *
Paroxysmal atrial fibrillation	86 (46)	27 (47)	44 (46)	15 (44)	0.97
Prior intervention					
Prior myocardial infarction	28 (15)	8 (14)	15 (16)	5 (15)	0.95
Prior percutaneous coronary intervention	70 (37)	23 (40)	37 (38)	10 (29)	0.58
Prior coronary artery bypass grafting	17 (9)	9 (16)	4 (4)	4 (12)	0.049 *
Scores					
CHADS_2_ score	3.5 ± 1.3	2.9 ± 1.2	3.7 ± 1.2	3.7 ± 1.2	<0.01 *
CHA_2_DS_2_-VASc score	5.1 ± 1.4	4.4 ± 1.3	5.4 ± 1.3	5.6 ± 1.4	<0.01 *
HAS-BLED score	2.9 ± 1.0	3.2 ± 1.0	2.9 ± 0.9	2.6 ± 1.0	0.034 *
CSHA	3.5 ± 1.1	3.4 ± 1.2	3.6 ± 0.9	4.2 ± 1.0	<0.01 *
High risk for falling-related bleeding	34 (18)	7 (12)	15 (16)	12 (35)	0.013 *
History of relevant bleeding					
Total	96 (51)	22 (45)	52 (54)	18 (53)	0.52
Intracranial	28 (15)	9 (16)	16 (17)	3 (9)	0.54
Gastrointestinal	46 (25)	9 (16)	26 (27)	11 (32)	0.14
Respiratory	3 (2)	2 (4)	1 (1)	0 (0)	0.37
Other	22 (12)	7 (12)	10 (10)	5 (15)	0.80
Transthoracic echocardiography					
Left atrial diameter (mm)	46.2 ± 8.2	46.4 ± 8.8	44.9 ± 7.8	49.5 ± 7.4	0.02 *
Left atrial volume index (mL/m^2^)	60.2 ± 26.5	56.2 ± 27.0	60.1 ± 26.1	67.5 ± 26.5	0.21
Left ventricular end-diastolic diameter (mm)	48.1 ± 6.8	49.8 ± 7.4	47.3 ± 6.2	47.7 ± 7.4	0.09
Left ventricular ejection fraction (%)	59.6 ± 11.3	58.7 ± 12.1	60.0 ± 11.2	60.4 ± 10.1	0.72
Antiplatelet/anticoagulant therapy					
None	2 (1)	2 (3)	0 (0)	0 (0)	0.11
SAPT	0 (0)	0 (0)	0 (0)	0 (0)	-
DAPT	1 (1)	1 (2)	0 (0)	0 (0)	0.33
Single anticoagulant therapy	106 (56)	28 (48)	59 (61)	19 (56)	0.28
Warfarin	13 (7)	7 (12)	6 (6)	0 (0)	0.08
DOAC	93 (50)	21 (36)	53 (55)	19 (56)	0.05
SAPT and anticoagulant therapy	76 (40)	25 (43)	36 (38)	15 (44)	0.71
SAPT and Warfarin	31 (16)	16 (28)	10 (10)	5 (15)	0.02 *
SAPT and DOAC	45 (24)	9 (16)	26 (27)	10 (29)	0.19
DAPT and anticoagulant (triple therapy)	3 (2)	2 (3)	1 (1)	0 (0)	0.37

SAPT, Single Antiplatelet Therapy; DAPT, Dual Antiplatelet Therapy; DOAC, Direct Oral Anti Coagulants. * *p* < 0.05. Continuous variables were compared by unpaired *t*-test. Categorical variables were compared by Fisher’s exact test.

**Table 2:** Procedural characteristics and post-procedural medications.

	Total (N = 188)	<75 Years (N = 58)	75–84 Years (N = 96)	≥85 Years (N = 34)	*p* Value
General procedure data					
Procedure success	187 (99)	58 (100)	96 (100)	33 (97)	0.10
General anesthesia	188 (100)	58 (100)	96 (100)	34 (100)	1.0
Transesophageal echocardiography	188 (100)	58 (100)	96 (100)	34 (100)	1.0
Concomitant procedure	23 (12)	7 (12)	11 (11)	5 (15)	0.89
Procedure-related data					
Anesthesia time (min)	122 ± 42	128 ± 47	118 ± 35	124 ± 51	0.33
Fluoroscopy duration (min)	18 ± 14	19 ± 16	17 ± 12	20 ± 15	0.37
Procedure time (min)	59 ± 32	61 ± 31	58 ± 30	60 ± 37	0.82
Contrast volume (mL)	54 ± 32	53 ± 27	56 ± 36	52 ± 27	0.78
TEE findings					
LAA peak velocity (cm/min)	29 ± 18	33 ± 24	28 ± 14	26 ± 14	0.22
SEC grade	2.4 ± 1.1	2.3 ± 1.1	2.5 ± 1.1	2.5 ± 0.9	0.52
LAA ostium diameter					
0 degree (mm)	20.7 ± 3.9	20.7 ± 4.0	20.7 ± 4.2	21.1 ± 3.0	0.84
45 degrees (mm)	19.8 ± 3.6	19.5 ± 3.6	19.9 ± 3.9	20.1 ± 2.8	0.75
90 degrees (mm)	20.6 ± 4.0	20.5 ± 3.6	20.4 ± 4.5	21.3 ± 3.1	0.53
135 degrees (mm)	22.9 ± 3.9	22.6 ± 3.9	22.8 ± 4.1	23.9 ± 3.5	0.25
Device					
Implanted device WATCHMAN 2.5/FLX	48/139	22/36	18/78	8/25	-
WATCHMAN 2.5 size 21/24/27/30/33 mm	2/1/11/11/23	21/0/27/3/11	1/1/4/5/7	0/0/0/3/5	-
WATCHMAN FLX size 20/24/27/31/35 mm	5/10/37/52/35	1/3/10/14/8	4/6/19/30/19	0/1/8/8/8	-
Device compression rate					
0 degree (%)	16.2 ± 5.9	16.2 ± 4.8	15.6 ± 5.7	18.0 ± 7.5	0.11
45 degrees (%)	16.8 ± 5.4	17.5 ± 5.5	16.2 ± 5.2	17.6 ± 5.9	0.26
90 degrees (%)	16.1 ± 6.3	17.3 ± 7.3	15.0 ± 5.4	17.3 ± 6.4	0.04 *
135 degrees (%)	15.1 ± 5.1	15.6 ± 5.1	14.3 ± 4.9	16.5 ± 5.7	0.09
Peri-device leak					
<3 mm	13 (7)	2 (3)	10 (10)	1 (3)	0.16
3–5 mm	0 (0)	0 (0)	0 (0)	0 (0)	-
>5 mm	0 (0)	0 (0)	0 (0)	0 (0)	-
Medications at index discharge					
None	1 (1)	1 (2)	0 (0)	0 (0)	0.33
SAPT only	0 (0)	0 (0)	0 (0)	0 (0)	-
DAPT only	0 (0)	0 (0)	0 (0)	0 (0)	-
Single anticoagulant therapy	49 (26)	11 (19)	28 (29)	10 (29)	0.34
Warfarin	4 (2)	1 (2)	2 (2)	1 (3)	0.93
DOAC	45 (24)	10 (17)	26 (27)	9 (26)	0.38
SAPT and anticoagulant therapy	136 (72)	45 (78)	67 (70)	24 (71)	0.56
SAPT and Warfarin	39 (21)	22 (38)	13 (14)	4 (12)	<0.01 *
SAPT and DOAC	97 (52)	23 (40)	54 (56)	20 (59)	0.09
DAPT and anticoagulant (triple therapy)	2 (1)	1 (2)	1 (1)	0 (0)	0.74

SAPT, Single Antiplatelet Therapy; DAPT, Dual Antiplatelet Therapy; DOAC, Direct Oral Anti Coagulants. * *p* < 0.05. Continuous variables were compared by unpaired *t*-test. Categorical variables were compared by Fisher’s exact test.

**Table 3:** Transesophageal echocardiography findings and medications at 45-day follow-up.

	Total (N = 188)	<75 Years (N = 58)	75–84 Years (N = 96)	≥85 Years (N = 34)	*p* Value
TEE parameter					
Procedure completion	163 (87)	48 (83)	85 (89)	30 (88)	0.81
Device compression rate					
0 degree (%)	13.5 ± 5.8	13.1 ± 6.6	13.7 ± 5.6	13.7 ± 5.5	0.81
45 degrees (%)	14.4 ± 6.2	14.0 ± 6.9	14.9 ± 6.2	13.6 ± 4.7	0.52
90 degrees (%)	13.7 ± 5.7	12.8 ± 5.5	14.2 ± 5.8	14.3 ± 5.5	0.37
135 degrees (%)	13.0 ± 5.2	13.0 ± 5.1	12.6 ± 5.7	14.2 ± 3.9	0.37
Peri-device leak					
<3 mm	34 (18)	12 (21)	16 (17)	6 (18)	0.82
3–5 mm	4 (2)	0 (0)	3 (3)	1 (3)	0.41
>5 mm	1 (1)	1 (2)	0 (0)	0 (0)	0.33
Device-related thrombosis	1 (1)	0 (0)	1 (1)	0 (0)	0.64
Medications					
None	17 (10)	6 (12)	7 (8)	4 (13)	0.68
SAPT	34 (20)	8 (15)	18 (20)	8 (26)	0.51
DAPT	89 (51)	29 (56)	45 (49)	15 (48)	0.86
Single anticoagulant therapy	26 (15)	7 (13)	16 (18)	3 (10)	0.35
Warfarin	2 (1)	1 (3)	0 (0)	1 (3)	0.87
DOAC	24 (14)	6 (12)	16 (18)	2 (6)	0.22
SAPT and anticoagulant therapy	8 (4)	2 (4)	5 (5)	1 (3)	0.81
SAPT and Warfarin	3 (2)	1 (2)	2 (2)	0 (0)	0.71
SAPT and DOAC	5 (3)	1 (2)	3 (3)	1 (3)	0.87
DAPT and anticoagulant (triple therapy)	0 (0)	0 (0)	0 (0)	0 (0)	-

SAPT, Single Antiplatelet Therapy; DAPT, Dual Antiplatelet Therapy; DOAC, Direct Oral Anti Coagulants. Continuous variables were compared by unpaired *t*-test. Categorical variables were compared by Fisher’s exact test.

**Table 4:** Transesophageal echocardiography findings and medications at 1-year follow-up.

	Total (N = 188)	<75 Years (N = 58)	75–84 Years (N = 96)	≥85 Years (N = 34)	*p* Value
TEE parameter					
Procedure completion	99 (53)	32 (55)	47 (49)	20 (59)	0.56
Device compression rate					
0 degree (%)	15.1 ± 6.6	16.7 ± 7.5	14.7 ± 6.2	13.4 ± 5.4	0.18
45 degrees (%)	16.1 ± 6.7	18.5 ± 7.3	15.0 ± 6.2	15.0 ± 6.0	0.049 *
90 degrees (%)	15.7 ± 6.0	17.5 ± 6.9	14.3 ± 5.4	16.0 ± 5.0	0.06
135 degrees (%)	13.6 ± 5.5	14.9 ± 5.3	12.5 ± 5.8	14.0 ± 5.0	0.15
Peri-device leak					
<3 mm	23 (23)	10 (31)	9 (19)	4 (20)	0.34
3–5 mm	4 (4)	2 (6)	2 (4)	0 (0)	0.54
>5 mm	1 (1)	0 (0)	1 (2)	0 (0)	0.58
Device-related thrombosis	6 (6)	2 (6)	3 (6)	1 (5)	0.98
Medications					
None	6 (5)	1 (3)	3 (6)	2 (8)	0.68
SAPT	91 (76)	24 (75)	45 (84)	22 (92)	0.25
DAPT	5 (4)	3 (9)	2 (4)	0 (0)	0.26
Single anticoagulant therapy	7 (6)	4 (13)	3 (6)	0 (0)	0.18
Warfarin	0 (0)	0 (0)	0 (0)	0 (0)	
DOAC	7 (6)	4 (13)	3 (6)	0 (0)	0.18
SAPT and anticoagulant therapy	0 (0)	0 (0)	0 (0)	0 (0)	-
SAPT and Warfarin	0 (0)	0 (0)	0 (0)	0 (0)	-
SAPT and DOAC	0 (0)	0 (0)	0 (0)	0 (0)	-
DAPT and anticoagulant (triple therapy)	0 (0)	0 (0)	0 (0)	0 (0)	-

SAPT, Single Antiplatelet Therapy; DAPT, Dual Antiplatelet Therapy; DOAC, Direct Oral Anti Coagulants. * *p* < 0.05. Continuous variables were compared by unpaired *t*-test. Categorical variables were compared by Fisher’s exact test.

**Table 5:** Clinical events for follow-up period.

	Total (N = 188)	<75 Years (N = 58)	75–84 Years (N = 96)	≥85 Years (N = 34)	*p* Value
Procedure related events within 7 days	1 (1)	0 (0)	0 (0)	1 (1)	0.10
Major procedure related complication	1 (1)	0 (0)	0 (0)	1 (1)	0.10
Death	0 (0)	0 (0)	0 (0)	0 (0)	-
Cerebrovascular events	0 (0)	0 (0)	0 (0)	0 (0)	-
Systemic embolism	0 (0)	0 (0)	0 (0)	0 (0)	-
Air embolism	0 (0)	0 (0)	0 (0)	0 (0)	-
Device migration	0 (0)	0 (0)	0 (0)	0 (0)	-
Device embolization	0 (0)	0 (0)	0 (0)	0 (0)	-
Any bleeding	1 (1)	0 (0)	0 (0)	1 (1)	0.10
Minor bleeding BARC 1-2	0 (0)	0 (0)	0 (0)	0 (0)	-
Major bleeding BARC 3-5	1 (1)	0 (0)	0 (0)	1 (1)	0.10
Pericardial effusion new onset	1 (2)	0 (0)	1 (1)	0 (0)	0.62
Clinically non-relevant	1 (2)	0 (0)	1 (1)	0 (0)	0.62
Clinically relevant	0 (0)	0 (0)	0 (0)	0 (0)	-
Vascular access site complication	0 (0)	0 (0)	0 (0)	0 (0)	-
Acute kidney injury	0 (0)	0 (0)	0 (0)	0 (0)	-
Non procedure related events within 7 days					
Death	1 (1)	0 (0)	1 (1)	0 (0)	0.46
Cardiovascular death	1 (1)	0 (0)	1 (1)	0 (0)	0.46
Cardiovascular event	1 (1)	0 (0)	1 (1)	0 (0)	0.46
Systemic embolism	0 (0)	0 (0)	0 (0)	0 (0)	-
Any bleeding	1 (1)	0 (0)	0 (0)	1 (3)	0.10
Minor bleeding BARC 1-2	0 (0)	0 (0)	0 (0)	0 (0)	-
Major bleeding BARC 3-5	1 (1)	0 (0)	0 (0)	1 (3)	0.10
All clinical events during follow-up					
Death	13 (7)	3 (5)	6 (6)	4 (12)	0.46
Cardiovascular death	4 (2)	0 (0)	3 (3)	1 (3)	0.41
Cerebrovascular event	3 (2)	0 (0)	3 (3)	0 (0)	0.24
Stroke	2 (1)	0 (0)	2 (2)	0 (0)	0.38
Ischemic stroke	2 (1)	0 (0)	2 (2)	0 (0)	0.38
Hemorrhagic stroke	0 (0)	0 (0)	0 (0)	0 (0)	-
Transient ischemic attack	2 (1)	0 (0)	1 (1)	1 (3)	0.42
Systemic embolism	1 (1)	1 (2)	0 (0)	0 (0)	0.32
Myocardial infarction	1 (1)	0 (0)	1 (1)	0 (0)	0.62
Any bleeding	10 (5)	1 (2)	7 (7)	2 (6)	0.33
Minor bleeding BARC 1-2	2 (1)	1 (2)	1 (1)	0 (0)	0.74
Major bleeding BARC 3-5	11 (6)	1 (2)	8 (7)	2 (6)	0.34
Pericardial effusion new onset	0 (0)	0 (0)	0 (0)	0 (0)	-
Follow up days	280 ± 181	286 ± 198	259 ± 174	328 ± 182	0.16

Variables were compared by Fisher’s exact test.

## Data Availability

Data are available from the corresponding authors upon reasonable requests.

## References

[1] Inoue H., Fujiki A., Origasa H., Ogawa S., Okumura K., Kubota I., Aizawa Y., Yamashita T., Atarashi H., Horie M. (2009). Prevalence of atrial fibrillation in the general population of Japan: An analysis based on periodic health examination. Int. J. Cardiol..

[2] Pearce L.A., Hart R.G., Halperin J.L. (2000). Assessment of three schemes for stratifying stroke risk in patients with nonvalvular atrial fibrillation. Am. J. Med..

[3] Pisters R., Lane D.A., Nieuwlaat R., De Vos C.B., Crijns H.J., Lip G.Y. (2010). A novel user-friendly score (HAS-BLED) to assess 1-year risk of major bleeding in patients with atrial fibrillation: The Euro Heart Survey. Chest.

[4] Connolly S.J., Ezekowitz M.D., Yusuf S., Eikelboom J., Oldgren J., Parekh A., Pogue J., Reilly P.A., Themeles E., Varrone J. (2009). Dabigatran versus warfarin in patients with atrial fibrillation. N. Engl. J. Med..

[5] Patel M.R., Mahaffey K.W., Garg J., Pan G., Singer D.E., Hacke W., Breithardt G., Halperin J.L., Hankey G.J., Piccini J.P. (2011). Rivaroxaban versus warfarin in nonvalvular atrial fibrillation. N. Engl. J. Med..

[6] Granger C.B., Alexander J.H., McMurray J.J., Lopes R.D., Hylek E.M., Hanna M., Al-Khalidi H.R., Ansell J., Atar D., Avezum A. (2011). Apixaban versus warfarin in patients with atrial fibrillation. N. Engl. J. Med..

[7] Giugliano R.P., Ruff C.T., Braunwald E., Murphy S.A., Wiviott S.D., Halperin J.L., Waldo A.L., Ezekowitz M.D., Weitz J.I., Špinar J. (2013). Edoxaban versus warfarin in patients with atrial fibrillation. N. Engl. J. Med..

[8] Ruff C.T., Giugliano R.P., Braunwald E., Hoffman E.B., Deenadayalu N., Ezekowitz M.D., Camm A.J., Weitz J.I., Lewis B.S., Parkhomenko A. (2014). Comparison of the efficacy and safety of new oral anticoagulants with warfarin in patients with atrial fibrillation: A meta-analysis of randomised trials. Lancet.

[9] Kato E.T., Goto S., Giugliano R.P. (2019). Overview of oral antithrombotic treatment in elderly patients with atrial fibrillation. Ageing Res. Rev..

[10] Blackshear J.L., Odell J.A. (1996). Appendage obliteration to reduce stroke in cardiac surgical patients with atrial fibrillation. Ann. Thorac. Surg..

[11] Holmes D.R., Kar S., Price M.J., Whisenant B., Sievert H., Doshi S.K., Huber K., Reddy V.Y. (2014). Prospective randomized evaluation of the Watchman Left Atrial Appendage Closure device in patients with atrial fibrillation versus long-term warfarin therapy: The PREVAIL trial. J. Am. Coll. Cardiol..

[12] Holmes D.R., Reddy V.Y., Turi Z.G., Doshi S.K., Sievert H., Buchbinder M., Mullin C.M., Sick P., Investigators P.A. (2009). Percutaneous closure of the left atrial appendage versus warfarin therapy for prevention of stroke in patients with atrial fibrillation: A randomised non inferiority trial. Lancet.

[13] Osmancik P., Herman D., Neuzil P., Hala P., Taborsky M., Kala P., Poloczek M., Stasek J., Haman L., Branny M. (2020). Left Atrial Appendage Closure Versus Direct Oral Anticoagulants in High-Risk Patients With Atrial Fibrillation. J. Am. Coll. Cardiol..

[14] Reddy V.Y., Holmes D., Doshi S.K., Neuzil P., Kar S. (2011). Safety of percutaneous left atrial appendage closure: Results from the watchman left atrial appendage system for embolic protection in patients with AF (PROTECT AF) clinical trial and the continued access registry. Circulation.

[15] Kar S., Doshi S.K., Sadhu A., Horton R., Osorio J., Ellis C., Stone J., Shah M., Dukkipati S.R., Adler S. (2021). Primary Outcome Evaluation of a Next-Generation Left Atrial Appendage Closure Device: Results from the PINNACLE FLX Trial. Circulation.

[16] Betts T.R., Grygier M., Nielsen Kudsk J.E., Schmitz T., Sandri M., Casu G., Bergmann M., Hildick-Smith D., Christen T., Allocco D.J. (2023). Real-world clinical outcomes with a next-generation left atrial appendage closure device: The FLXibility Post-Approval Study. Europace.

[17] Aonuma K., Yamasaki H., Nakamura M., Matsumoto T., Takayama M., Ando K., Hirao K., Goya M., Morino Y., Hayashida K. (2020). Efficacy and safety of left atrial appendage closure with WATCHMAN in Japanese nonvalvular atrial fibrillation patients—Final 2-year follow-up outcome data from the SALUTE Trial. Circ. J..

[18] Nogami A., Kurita T., Kusano K., Goya M., Shoda M., Tada H., Naito S., Yamane T., Kimura M., Shiga T. (2022). JCS/JHRS 2021 Guideline Focused Update on Non-Pharmacotherapy of Cardiac Arrhythmias. Circ. J..

[19] Ono K., Iwasaki Y., Shimizu W., Akao M., Ikeda T., Ishii K., Inden Y., Kusano K., Kobayashi Y., Koretsune Y. (2022). JCS/JHRS 2020 Guideline on Pharmacotherapy of Cardiac Arrhythmias. Circ. J..

[20] Yamashita Y., Hamatani Y., Esato M., Chun Y.H., Tsuji H., Wada H., Hasegawa K., Abe M., Lip G.Y.H., Akao M. (2016). Clinical Characteristics and Outcomes in Extreme Elderly (Age ≥ 85 Years) Japanese Patients With Atrial Fibrillation: The Fushimi AF Registry. Chest.

[21] Sellers M.B., Newby L.K. (2011). Atrial fibrillation, anticoagulation, fall risk, and outcomes in elderly patients. Am. Heart J..

[22] Davies L.E., Spiers G., Kingston A., Todd A., Adamson J., Hanratty B. (2020). Adverse Outcomes of Polypharmacy in Older People: Systematic Review of Reviews. J. Am. Med. Dir. Assoc..

[23] Yamashita T., Suzuki S., Inoue H., Akao M., Atarashi H., Ikeda T., Okumura K., Koretsune Y., Shimizu W., Tsutsui H. (2022). Two-year outcomes of more than 30,000 elderly patients with atrial fibrillation: Results from the All Nippon AF In the Elderly (ANAFIE) Registry. Eur. Heart J. Qual. Care Clin. Outcomes.

[24] Boersma L.V., Ince H., Kische S., Pokushalov E., Schmitz T., Schmidt B., Gori T., Meincke F., Protopopov A.V., Betts T. (2017). Efficacy and safety of left atrial appendage closure with WATCHMAN in patients with or without contraindication to oral anticoagulation: 1-Year follow-up outcome data of the EWOLUTION trial. Heart Rhythm..

[25] Price M.J., Slotwiner D., Du C., Freeman J.V., Turi Z., Rammohan C., Kusumoto F.M., Kavinsky C., Akar J., Varosy P.D. (2022). Clinical Outcomes at 1 Year Following Transcatheter Left Atrial Appendage Occlusion in the United States. JACC Cardiovasc. Interv..

[26] Fukuda N., Imamura T., Tanaka S., Kataoka N., Ushijima R., Ueno H., Kinugawa K. (2022). Comparison in Short-Term Safety and Efficacy between New-Generation WATCHMAN FLX and Conventional WATCHMAN 2.5 for Percutaneous Left Atrial Appendage Closure. J. Clin. Med..

[27] Ryuzaki S., Kondo Y., Nakano M., Nakano M., Kajiyama T., Ito R., Kitagawa M., Sugawara M., Chiba T., Yoshino Y. (2023). Antithrombotic Regimen After Percutaneous Left Atrial Appendage Closure—A Real-World Study. Circ. J..

[28] Simard T., Jung R.G., Lehenbauer K., Piayda K., Pracon R., Jackson G.G., Flores Umanzor E., Faroux L., Korsholm K., Chun J.K.R. (2021). Predictors of Device-Related Thrombus Following Percutaneous Left Atrial Appendage Occlusion. J. Am. Coll. Cardiol..

